# Ulcers a Cause of Bright’s Disease

**Published:** 1871-05

**Authors:** 


ULCERS A CAUSE OF BRIGHT'S DISEASE.—Professor Fischer, of Breslau, has shown that chronic ulcers of the legs, if allowed to persist unhealed, invariably lead to amyloid degeneration of the kidney. Hence the cure of such ulcers becomes a matter of great importance. Dr. Fischer recommends Langenbeck's continuous bath as the best treatment.—*Journal of Cutaneous Medicine.*

